# Impact of the Paediatric Intensive Care Outreach Network service on mortality within intensive care

**DOI:** 10.1186/cc11112

**Published:** 2012-03-20

**Authors:** K Sadasivam, S Skellett

**Affiliations:** 1 Great Ormond Street Hospital for Children, London, UK

## Introduction

We audited the mortality rate by admission source in our paediatric ICU, a paediatric tertiary referral centre, from 2004 to 2008 and found that the group of emergency unplanned internal admissions had a higher Standardised Mortality Ratio (SMR) of 1.55 compared to a SMR of 1.00 overall for patients admitted to the paediatric ICU. This was in keeping with data from other large paediatric centres [1]. The reasons for the increased mortality for this internal group were not clear and possibly multifactorial. To help address this, a Paediatric Intensive Care Outreach Network (ICON) team was developed and introduced in September 2009.

## Methods

A before-and-after study design was used to determine differences in percentage of admissions, mortality rate and SMR. Data were collected using the PICANet database for emergency unplanned internal admissions before (August 2004 to August 2008) and after implementation of the ICON team (August 2009 to February 2011). PICANet is a national database that audits all paediatric intensive care admissions in the UK [2].

## Results

A total of 3,629 admissions during a 4-year period pre ICON (August 2004 to August 2008) and 1,446 admissions during 18 months post ICON (August 2009 to February 2011) were audited. Following the introduction of ICON the percentage of unplanned admissions fell from 36.68% to 22.9%. These patients also had a lower mortality rate (14.57% vs. 9.36%) and the SMR decreased from 1.55 to 1.35.

## Conclusion

Our data show that the mortality rate has decreased since the introduction of ICON although a confounding factor could be a concurrent decreased crude mortality rate (5.5% in 2003 to 2004 vs. 4.2% 2008 to 2010) in all paediatric intensive care patients in the UK [2]. Despite this we believe that ICON is a significant contributing factor in identifying and rescuing patients on the wards before further significant deterioration requiring intensive care. Further ongoing audit is required.
